# Including the voice of paediatric patients: Cocreation of an engagement game

**DOI:** 10.1111/hex.13530

**Published:** 2022-06-24

**Authors:** Lorynn Teela, Lieke E. Verhagen, Mariken P. Gruppen, Maria J. Santana, Martha A. Grootenhuis, Lotte Haverman

**Affiliations:** ^1^ Child and Adolescent Psychiatry and Psychosocial Care, Emma Children's Hospital, Amsterdam UMC University of Amsterdam Amsterdam The Netherlands; ^2^ Mental Health Amsterdam Public Health Amsterdam The Netherlands; ^3^ Child development Amsterdam Reproduction and Development Amsterdam The Netherlands; ^4^ Department of General Pediatrics, Emma Children's Hospital, Amsterdam UMC University of Amsterdam Amsterdam The Netherlands; ^5^ Department of Community Health Sciences, Cumming School of Medicine University of Calgary Calgary Alberta Canada; ^6^ Department of Pediatrics, Cumming School of Medicine University of Calgary Calgary AB Canada; ^7^ Psychosocial Research and Care Innovation Princess Maxima Center for Pediatric Oncology Utrecht The Netherlands

**Keywords:** adolescent, codevelopment, patient engagement, patient participation, paediatrics, shared decision‐making, user‐centred design

## Abstract

**Background:**

Engaging patients in health care, research and policy is essential to improving patient‐important health outcomes and the quality of care. Although the importance of patient engagement is increasingly acknowledged, clinicians and researchers still find it difficult to engage patients, especially paediatric patients. To facilitate the engagement of children and adolescents in health care, the aim of this project is to develop an engagement game.

**Methods:**

A user‐centred design was used to develop a patient engagement game in three steps: (1) identification of important themes for adolescents regarding their illness, treatment and hospital care, (2) evaluation of the draft version of the game and (3) testing usability in clinical practice. Adolescents (12–18 years) were engaged in all steps of the development process through focus groups, interviews or a workshop. These were audio‐recorded, transcribed verbatim and analysed in MAXQDA.

**Results:**

(1) The important themes for adolescents (*N* = 15) were included: visiting the hospital, participating, disease and treatment, social environment, feelings, dealing with staff, acceptation, autonomy, disclosure and chronically ill peers. (2) Then, based on these themes, the engagement game was developed and the draft version was evaluated by 13 adolescents. Based on their feedback, changes were made to the game (e.g., adjusting the images and changing the game rules). (3) Regarding usability, the pilot version was evaluated positively. The game helped adolescents to give their opinion. Based on the feedback of adolescents, some last adjustments (e.g., changing colours and adding a game board) were made, which led to the final version of the game, *All Voices Count*.

**Conclusions:**

Working together with adolescents, *All Voices Count*, a patient engagement game was developed. This game provides clinicians with a tool that supports shared decision‐making to address adolescents' wishes and needs.

**Patient or Public Contribution:**

Paediatric patients, clinicians, researchers, youth panel of Fonds NutsOhra and patient associations (Patient Alliance for Rare and Genetic Diseases, Dutch Childhood Cancer Organization) were involved in all phases of the development of the patient engagement game—from writing the project plan to the final version of the game.

## 1 INTRODUCTION

Nowadays, engaging patients in health care is central to improving health outcomes that matter to them.^1^, ^2^ In health care, the concept of patient engagement applies to involving patients in decisions about their daily clinical care while addressing patients' wishes and needs.^1^, ^2^, ^3^, ^4^, ^5^, ^6^ In day‐to‐day care, this means that patients are informed about the choice in treatment options to make decisions that are aligned with patients' preferences.^3^, ^4^ In addition, efforts are increasingly being made to engage patients at a broader level of health care, including the level of the hospital organization, research and policy.^4^, ^6^, ^7^ The extent to which patients influence the decision‐making processes varies from consultation to active partnership—and everything in between.^4^, ^8^ For example, studies showed the involvement of adolescent patients in designing a youth‐friendly ward and identifying their preferences regarding a study design or measurement of outcomes.^9^, ^10^, ^11^, ^12^ In whatever shape, patient engagement benefits both patients and organizations: It not only improves the quality of care but also improves patient experience and self‐confidence, resulting in better health outcomes and higher inclusion rates in research.^2^, ^3^, ^7^, ^13^

Although the benefits of patient engagement are beyond dispute, clinicians and researchers still struggle with engaging patients in health care and research.^14^, ^15^ Mentioned reasons are that clinicians doubt whether patients are knowledgeable,^16^ involving patients is time consuming^16^, ^17^ and scheduling meetings with groups of patients is difficult.^18^ Involving paediatric patients seems to be especially challenging,^19^, ^20^ as the competence of children to participate is even more questioned.^21^, ^22^, ^23^ Also, the involvement of parents makes the process of engaging complex because of the paternalist approach to care.^13^, ^21^, ^22^ Finally, clinicians have little experience in *how* to involve children in matters pertaining to health care.^22^

Boenink et al.^24^ developed a tool to engage adults in translational research, *The Voice of Patients*. With this card game, patients can reflect on various topics regarding biomedical research. The uptake of the tool was positive, exceeding expectations from both patients and researchers.^24^ However, an engagement tool for children and adolescents is missing, but would be valuable to facilitate engaging paediatric patients. Thus, to fill in this gap, the aim of this study is to develop a patient engagement game for adolescents with a chronic condition that can be used by clinicians and researchers to incorporate what matters to paediatric patients in hospital care, research and policy. This game was developed in cocreation with adolescents through three different steps: (1) identification of the most important themes for adolescents in health care and finding out preferences for patient engagement, (2) development and evaluation of the game and (3) test the game usability in clinical practice.

## 2 Methods

A user‐centred design, as described in the literature by Gulliksen et al.,^25^ was used. Key principles of an user‐centred design include *user‐focused* and *active user involvement* throughout the entire development process. These principles were guaranteed by actively involving all representative users, including adolescents with a chronic condition, clinicians, researchers, the Patient Alliance for Rare and Genetic Diseases (VSOP), the Dutch Childhood Cancer Organization (VKN) and a youth panel of Fonds NutsOhra (FNO). This youth panel consists of adolescents with a chronic condition, who contributed with ideas and suggestions to several projects aiming to improve social engagement in health care. All representative users were involved in all phases of the design process—from writing the project plan to the final version of the game. Other principles, such as *prototyping* and *evaluate use in context*, were applied by developing, testing and continuously adapting the draft versions of the game. In addition, the draft versions were tested at every stage of the development process with the end‐users in a real‐life context. For the development and design of the game, we collaborated with design agency Studio Dam (*professional attitude—* www.studiodam.nl).

**Figure 1:**
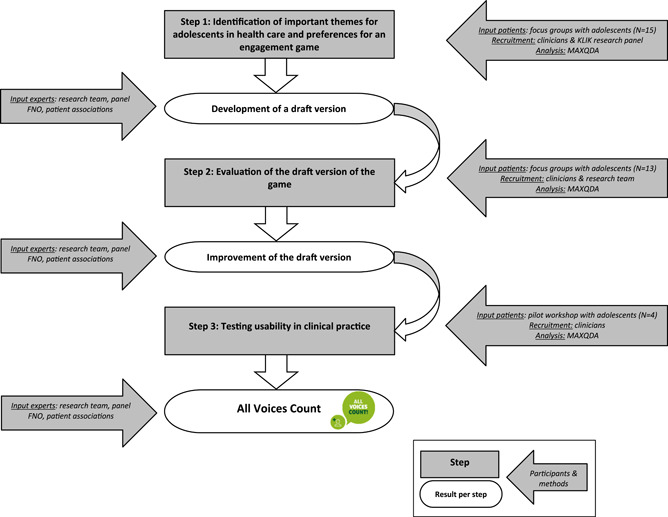
The development process of the game *All Voices Count*.

The development of the patient engagement game was an iterative process consisting of three steps (Figure 1):

1. Identification of important themes for adolescents regarding their illness, treatment, hospital care and influence on daily life and preferences for an engagement game. The identified themes will serve as a starting point for the development of the patient engagement game.
2. Evaluation of the draft version of the game.
3. Testing usability in clinical practice.

Each step resulted in the development/improvement of the game and provided input for the next step.

### 2.1 Step 1: Identification of important themes for adolescents in health care and preferences for an engagement game

For the first step, adolescents (12–18 years) with a chronic condition, under treatment at the Emma Children's Hospital Amsterdam UMC, were invited to participate in 60‐min focus groups and individual interviews (30–60 min) to identify important themes for adolescents in health care. Adolescents were recruited for this study by their clinician in June and July 2017. Additionally, patients who were part of the research panel of the KLIK patient‐reported outcome measures (PROM) portal (www.hetklikt.nu) were approached by the research team.^26^ The KLIK research panel consists of patients who have indicated, during registration for the KLIK PROM portal, that they would like to be approached for research projects in the Emma Children's Hospital.

During the focus groups and interviews, the elicitation technique ‘Complain and Cheer wall’^27^ was used. Adolescents were invited to write down things they did not like about living with a chronic condition, their treatment and the hospital care on the ‘Complain wall’ and things they did like on the ‘Cheer wall’. Thereafter, topics were discussed and grouped into themes by the adolescents and discussion leader following the Metaplan method (a workshop technique used to form a common understanding).^28^ In addition, adolescents were asked for their opinion regarding the development of a patient engagement card game. Data collection was continued until data saturation was reached. Data saturation was considered reached when no new themes emerged during the analyses of the focus groups. The result of these focus groups and interviews was a list of important themes for adolescents in health care and their preferences for an engagement game, which was used for the development of the first draft version of the game.

### 2.2 Step 2: Evaluation of the draft version of the game

In the second step, the first draft of the game was tested and evaluated with adolescents in a fictional context. Again, adolescents (12–18 years) with a chronic condition, under treatment at the Emma Children's Hospital Amsterdam UMC, were invited to participate in these focus groups and individual interviews. Adolescents were recruited from November 2017 till January 2018 in three ways: (1) participating adolescents in Step 1 were asked to participate again in this evaluation, (2) adolescents were approached by their clinician or (3) adolescents could sign up themselves after reading an information leaflet in the waiting room.

During 90‐min focus groups and interviews (45–60 min), the game was played with the adolescents in a fictional context (opinion about the use of patient‐reported outcome measures [PROMs]). Afterwards, adolescents were asked to evaluate the engagement game with the use of traffic light colours. Adolescents were invited to write down what they liked about the game (green), which parts of the game they were doubting about (yellow) and which parts of the game they did not like (red). Adolescents were asked to provide feedback on both the content and layout of the game. These topics were discussed, and adolescents were asked for suggestions for improvement and their opinion about specific aspects of the game (i.e., completeness of the included themes, desired game time and the use of photos or clip‐arts). Data collection was continued until data saturation was reached. The result of these focus groups and interviews was a list of improvements for the game, which was used to develop a pilot version of the game.

### 2.3 Step 3: Testing usability in clinical practice

The third step involved usability testing of the pilot version of the game. In this field test, a pilot workshop was held with patients from the educational facility (educational support service for patients and their parents) of the Emma Children's Hospital Amsterdam UMC. Participating patients were recruited via clinicians of the educational facility in April 2018.

During the 90‐min pilot workshop, the game was played with the adolescents to answer a question from the educational facility: ‘What can the educational facility do (even more) for you to ensure that things go even better at school?’. Afterwards, adolescents were asked to evaluate the engagement game using traffic light colours and were asked for suggestions for improvement. The result was that insight was gained into the usability of the engagement game and a list of improvements was obtained for the game, which was used to develop a final version of the game.

*For all steps*, participating adolescents and their parents (for adolescents <16 years) provided written informed consent and a sociodemographic questionnaire (i.e., age, gender, type of chronic disease) was completed by parents. Participants received a gift card (with an amount of 10 euro) and compensation for their travel expenses. Additionally, all focus groups, interviews and the pilot workshop were audio‐recorded, transcribed verbatim and analysed in MAXQDA^29^ following the methodology for thematic analysis.^30^ The focus groups, interviews and pilot workshop were conducted by two members of the research team. These members have been trained in conducting qualitative research.

## 3 RESULTS

The results are reported for every step of the development process. In total, 23 adolescents (range: 12–18 years, 57% female) participated in the cocreation of the patient engagement game, of whom nine adolescents participated in multiple steps (Table 1).

**Table 1:** Sociodemographic characteristics of participants in every step of the development process.

	Step 1: Identification of important themes (*N* = 15)	Step 2: Evaluating draft version (*N* = 13)^a^	Step 3: Usability testing (*N* = 4)
	*M* (range)	*M* (range)	*M* (range)
Age (years)	15.0 (12–18)	15.5 (13–18)	14.5 (13–16)
	%	%	%
Gender (female)	60	61.5	50
Type of chronic disease	%	%	%
Cancer	20	23.0	100
Sickle cell disease	26.6	15.4	
Cystic fibrosis	13.3	15.4	
Juvenile idiopathic arthritis	13.3	15.4	
Kidney disease		15.4	
Chronic eczema	6.7	7.7	
Asthma		7.7	
Chronic pain	6.7		
Crohn's disease	6.7		
Muscular diseases	6.7		

^a^ Nine adolescents also participated in Step 1.

### 3.1 Step 1: Identification of important themes for adolescents in health care and preferences for an engagement game

In total, 15 adolescents (mean age: 15.0 years, range 12–18 years, 60% female) participated in four focus groups and four interviews (Table 1). Ten major themes for adolescents regarding their illness, treatment and hospital care were identified: visiting the hospital, participating, disease and treatment, social environment, feelings, dealing with staff, acceptation, autonomy, disclosure and chronically ill peers (Table 2). Most of the adolescents liked the idea of a patient engagement card game. A few adolescents mentioned that they would prefer an online game. Based on the identified themes, a draft version of the game was developed by design agency Studio Dam, in consultation with the research team consisting of psychologists and medical doctors, representatives of the youth panel of FNO and the patient associations (VSOP, VKN).

**Table 2:** Overview of the identified themes and associated quotes (Step 1), and the adjusted names for the engagement game (Step 2).

Identified themes	Name in the game	Quotes
Visiting the hospital	My hospital	‘I like the shops in the hospital’.
		‘The things they organize for patients are very nice so I won't get bored’.
Participating	I can (not) do this	‘When I'm admitted to the hospital, it feels like I'm missing a few weeks of my life’.
		‘I miss normal things, like hanging out with friends or going to school’.
Disease & treatment	My disease & treatment	‘I don't like that I am getting tired due to the antibiotics that I have to take’.
		‘They made some mistakes in my treatment, for example once I got too much morphine’.
Social environment	Me & others	‘It is nice when people sympathize, because then I know that there are people who care about me’.
		‘My friends always tell me that they can't imagine how it is to have juvenile arthritis’.
Feelings	I feel this	‘I was always really afraid that something was wrong when I got the results back’.
		‘It feels very lonely when you think about your friends who are not sick’.
Dealing with staff	The people in my hospital	‘Sometimes doctors talk for hours and ask a lot of questions. I don't want that’.
		‘I like doctors and nurses to be honest, don't tell me that it won't hurt if it will hurt’.
Acceptation	I am okay	‘I just want to be normal, I want to participate in class and not feel tired or sick’.
		‘I feel a bit of an outsider’.
Autonomy	I do (not do) it myself	‘Because I am young, they don't take me seriously. That is annoying’.
		‘I take care of myself’.
Disclosure	Talk about it	‘I'm not willing to tell my life story’.
		‘I like to share my story’.
Chronically ill peers	Just like me	‘They understand me better than my normal friends’.
		‘Kids who are sick too are more interested in my illness’.

*Note*: All quotes were translated into English.

### 3.2 Step 2: Evaluation of the draft version of the game

**Figure 2:**
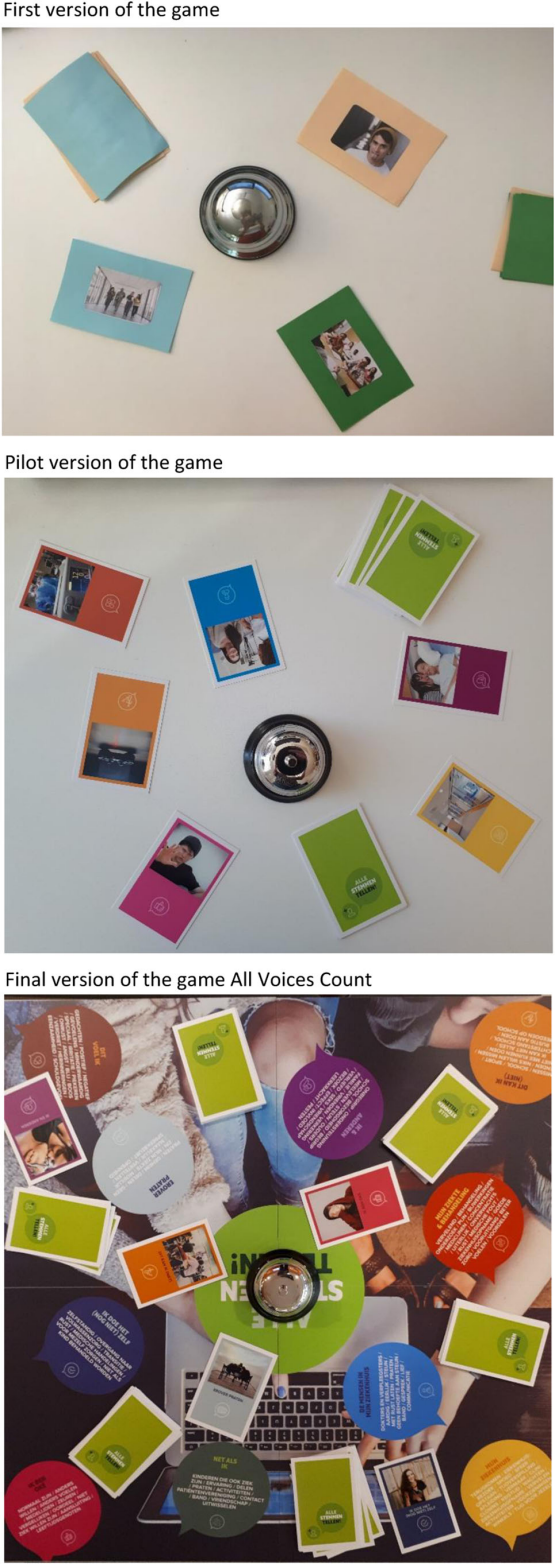
Overview of the different versions of the engagement game during the development process.

The opinion of 13 adolescents (mean age: 15.5 years, range: 13–18 years, 61.5% female, Table 1) was asked about the draft version (Figure 2) of the engagement game in three focus groups and five interviews. Overall, the adolescents were positive about the game as it gave them the opportunity to get involved and it helped them to express their views. They indicated that the use of themes and images made it easier for most adolescents to associate and think of other topics to express their opinion on. The game element was appreciated; it was fun, exciting and motivates competition. Furthermore, the design of the game was attractive and it was easy to play. Suggestions for improvement were about the explanation of the game and the images on the playing cards. Although adolescents mentioned a preference for images rather than clip‐arts, the majority mentioned that the images on the cards were not clear and that the persons in the images were too old, preventing them from relating to the depicted situation. Therefore, we adapted the images and tried to match the age group of 12–18 years. A few adolescents suggested the addition of keywords about the situations to the playing cards, but we decided not to because it can reduce the possibility of free association. Furthermore, adolescents indicated that a map with an overview of the themes would be helpful. In addition, a few adolescents made some suggestions to improve the design of the game, for example, changing the rules of the game or creating an online version. These suggestions were discussed with the research team, the design agency and the representative of the youth panel. We decided to change the images, the layout of the playing cards, the rules of the game and the game explanation, and we added an personal overview card of the themes and associated subthemes. This card clarifies to adolescents what kinds of subthemes are related to the specific themes (Table 3). Finally, we discussed the naming of the themes within the project group, as we noticed that the naming did not always match the perception of adolescents. We decided to rename the themes to make them more appealing and understandable for adolescents: my hospital, I can (not) do this, my disease & treatment, me & others, I feel this, the people in my hospital, I am okay, I do (not do) it myself, talk about it and just like me (Table 2).

**Table 3:** Overview of the feedback of adolescents and the adjustments that were made to the game (Steps 2 and 3).

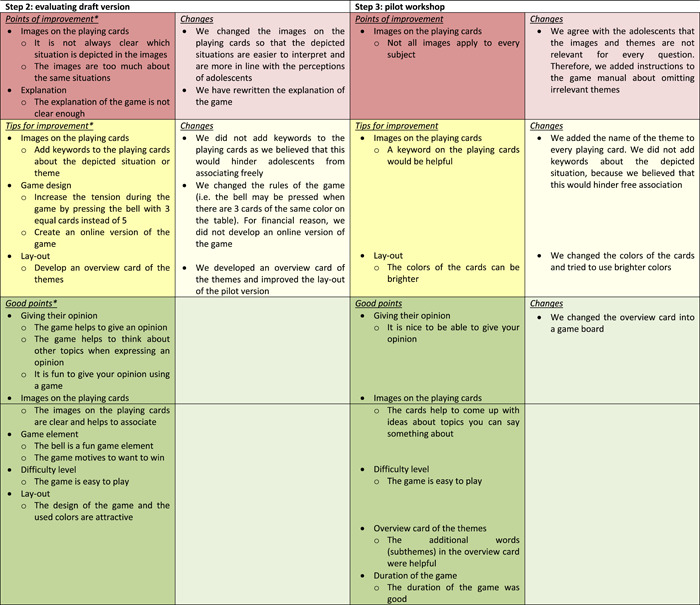

^a^ Mentioned by adolescents in two or more focus groups/interviews.

### 3.3 Step 3: Testing usability in clinical practice

The pilot version (Figure 2) of the game was tested for usability by four patients (mean age: 14.5 years, range: 13–16 years, 50% female; Table 1) in clinical practice. During this pilot workshop, the adolescents gave their opinion about a question of the educational facility, and a report on this workshop has been presented to the education facility to help them improve their daily clinical care. At the end of the workshop, the adolescents gave their opinion about the engagement game. All adolescents were enthusiastic about the game and enjoyed giving their opinion. Although it was difficult for some adolescents to give an opinion on all themes, the cards helped adolescents to come up with ideas about topics to talk about. The adolescents mentioned that not all themes were applicable for the educational facility. Therefore, we added some instructions to the game manual for professionals about the selection of themes and the minimum number of themes to be included in the game (Table 3). Furthermore, adolescents suggested the addition of keywords about the situations to the playing cards. In consultation with the research team, we decided to add the theme name to all playing cards. Adolescents evaluated the overview card of the themes (as developed and added after Step 2) positively; however, we noticed that these personal maps were distracting and reduced the group feeling. By introducing the themes on a game board (Table 3), the focus of all players is on the game, and they are invited to help each other as not all words are visible from every corner. Finally, we changed the colours (brighter colours) of the themes and cards and after that a final version of the engagement game, called *All Voices Count*, was ready (Box 1). To support clinicians with the use of *All Voices count*, we developed a game manual, a website (www.allestemmentellen.nl) and a training.

**Game rules *All Voices Count***

**All Voices Count**

*All Voices Count* is a patient engagement game that helps clinicians to engage adolescents (12–18 years) with a chronic condition in their hospital care, research or policy. The game is played at the initiative of a clinician and before the meeting of All Voices Count the clinician prepares a 2–4 min video pitch, in which the question on which the clinician would like to hear the opinion of adolescents is presented to the participants. This question is the central topic of the meeting.

*Examples of questions*:

1. *What do you think of this new treatment?*
2. *What does it mean for you to have a coagulation disease and how can we help you?*

All Voices Count is accompanied by a game leader. The clinician (of the adolescents) is not present during the meeting to avoid that adolescents are being inhibited in expressing their opinion.

**The course of the game**

The game starts with a short introduction in which the participants get to know each other and watch the pitch of the clinician. Then, the game starts:

1. In turns, players turn over the top card of their deck and place it face upwards anywhere on the game board. Each subsequent card is placed anywhere on the game board, so that all cards played remain visible for every player.
2. When three cards of the same colour (same theme) are visible on the game board, every player may press the bell. The player who presses the bell first wins all the cards of the same colour (same theme) that are visible on the game board.
3. The player who wins the cards may give his or her opinion first on the question posed by the clinician, regarding the theme of the cards won. To get ideas, the player may look at the pictures on the cards won or the words belonging to the theme on the game board. All other players are allowed to react and give their own opinion.
4. When the players are done talking about the cards won, a new round starts.

The game ends when all cards have been played or if the fixed play time has elapsed. The player with the most cards won is the winner of the game.

*Additional remarks*

1. All Voices Count can be played with 3–6 adolescents.
2. The game leader plays an important role in steering the meeting (e.g., to make sure that the question of the clinicians remains the central topic and that all participants get the opportunity to express their opinion) and to help the participants with expressing their opinion whenever they are struggling with this. The game leader does not express his or her own opinion.
3. Not all themes are relevant for every question. The game leader may decide to remove irrelevant themes. All Voices Count can be played with a minimum of five themes.

## 4 DISCUSSION

Working together with adolescents, we co‐developed and tested the usability of a paediatric patient engagement game, *All Voices Count*. This resulted in a valued tool that makes it easier for clinicians to include the input from paediatric patients in the decision‐making process of hospital care, research and policy. Overall, adolescents were pleased with *All Voices Count* as it enables them to express their opinion and experiences regarding different topics in health care more easily.

The first step in developing *All Voices Count* was to identify important themes for adolescents regarding their chronic condition, treatment and hospital care. Further development of the game was based on these themes to connect to the perception of adolescents with a chronic condition. The identified themes were aligned with previous studies,^31^, ^32^, ^33^ in which participation in daily life, being normal, treatment, social environment and communication about their disease were also seen as important themes by adolescents with other conditions. This corroboration showed that adolescents, regardless of their chronic condition, face similar difficulties and supportive factors.

Since the development of *All Voices Count*, the game has been used to include the opinion of adolescents in several projects in our hospital. For example, *All Voices Count* was used during the development of an International Core Outcome Set for acute simple appendicitis in children.^34^ With the use of *All Voices Count*, important outcomes for adolescents in determining the effectiveness of treatment were identified (*What do you think is important to know to make an informed choice between two treatments for appendicitis?*) and subsequently prioritized. In addition, we are planning to use *All Voices Count* for questions from the physiotherapist of the department of oncology (*How can we make exercising more fun for you during treatment?*) to improve daily hospital care, for questions from researchers and clinicians from the haematology department (*What does it mean for you, as a girl, to have a coagulation disease and how can we improve the care?*) and for questions from clinicians and policymakers from the paediatric surgery department (*How should the follow‐up programme look like and which themes should be discussed by the clinician?*) for setting up a new follow‐up programme. Other purposes for which the game could be used are within the Kids Advisory Board of the children's hospitals and to discuss new research ideas with adolescents while writing a grant proposal. Engaging patients in the development of new research projects is increasingly being mentioned as a requirement for research funding.^35^, ^36^

The next step is a further distribution and implementation of *All Voices Count* in other children's hospitals and rehabilitation centres in the Netherlands. Our goal is to bring *All Voices Count* to the attention of professionals working in different areas within the health care sector. We will therefore present *All Voices Count* at international conferences and to policymakers and division boards of hospitals in the Netherlands. Furthermore, we train clinicians in how to use the game and in the way in which they can use the results obtained in their daily clinical care, research or policy. To be able to deploy the game widely, we recently translated the Dutch version of *All Voices Count* into English.

The strengths of the user‐centred design used in this study were that it provided insight into the perspective of the users and that it facilitated new ideas, so that it meets the needs of the users.^25^, ^37^ Especially, the input from adolescents was very valuable to us during the development process. Adolescents thought critically about the game and came up with valuable suggestions to improve the game. *All Voices Count* has been tested in a real‐life context, making it usable and appropriate to the cultural context, and it increases the chances of successful implementation.^25^, ^38^

Challenges or limitations in our user‐centred design were the degree of influence and control of the participants and the representativeness.^17^ While adolescents were involved throughout all phases of the development process, the research team included researchers, clinicians, representatives of a youth panel and patient associations, reviewed the final version. Regarding the representativeness, we invited adolescents with different chronic conditions to participate in this study. Now, during the evaluation of the pilot version of the game, only adolescents with cancer participated, which may have limited the representativeness of our study. However, this study showed that adolescents, regardless of their chronic condition, showed the same problems and supportive factors; therefore, we do not believe that this has influenced the results. Furthermore, earlier research showed that paediatric patients willing to participate in codesign studies tend to be more self‐confident, critical and assertive adolescents, which can further hinder representativeness.^17^, ^39^ Finally, we tried to include the same adolescents in several steps of the development process to give them the opportunity to be a part of the project and to hear their views on the changes that were made based on their feedback. The engagement of adolescents multiple times can have advantages, such as adolescents can express their views on the changes made to the game and are well informed, and disadvantages like adolescents can express views that are a bit more one‐sided, and fewer new ideas. During the different steps of the development process, we therefore included adolescents that did not participated in earlier steps as adolescents that participated in earlier steps.

Some barriers to engagement in this study included logistic difficulties related to travelling to the hospital, time constraints and difficulties in scheduling a meeting with a group of adolescents. These barriers have been mentioned by both adolescents and adults in other fields.^7^, ^18^, ^40^ Developing an online version of the game could potentially reduce these barriers, according to the adolescents in our study. Adolescents indicated that the advantages of an online game are that they do not have to visit the hospital, that it takes less time and that they can fit it more flexibly into their time schedule. For this reason, we would like to develop an online version of *All Voices Count* in the future.

## 5 CONCLUSION

In conclusion, we developed a patient engagement game called *All Voices Count*, working together with all stakeholders. This game lowers the barrier to include the voice of adolescents in decision‐making about hospital care, research and policy.

## AUTHOR CONTRIBUTIONS

Lotte Haverman conceived the study. Lorynn Teela and Lieke E. Verhagen carried out the focus groups and interviews and performed the qualitative analyses. The first draft of the manuscript was written by Lorynn Teela. Mariken P. Gruppen, Maria J. Santana, Martha A. Grootenhuis and Lotte Haverman handled the supervision. All authors critically revised the manuscript for intellectual content and approved the final version of the manuscript.

## CONFLICT OF INTEREST

The authors declare no conflict of interest.

## ETHICS STATEMENT

All procedures performed in this study were in accordance with the ethical standards of the international and/or national research committee (Medical Ethics Committee of the Amsterdam UMC – W17_068#17.086) and with the 1964 Helsinki declaration and its later amendments or comparable ethical standards. Informed consent was obtained from all individual participants included in this study.

## Data Availability

The data that support the findings of this study are available from the corresponding author upon reasonable request.
